# Supplementary material for: Pyruvate Dehydrogenase Kinase 1 inhibition mediated oxidative phosphorylation enhancement in cartilage promotes osteoarthritis progression
Source: BMC Musculoskelet Disord. 2023 Jul 20;24:597. doi: 10.1186/s12891-023-06585-6 (PMC10357736; doi:10.1186/s12891-023-06585-6)
Supplement: Supplementary file 1 — Additional file 1. [file 12891_2023_6585_MOESM1_ESM.zip › KEGG.Gsea.1653622432464/KEGG_BASAL_CELL_CARCINOMA.html]

Details for gene set KEGG\_BASAL\_CELL\_CARCINOMA[GSEA]

|  || Dataset | 55457\_collapsed\_to\_symbols.cls.cls #OA\_versus\_normal.cls.cls #OA\_versus\_normal\_repos |
| Phenotype | cls.cls#OA\_versus\_normal\_repos |
| Upregulated in class | normal |
| GeneSet | KEGG\_BASAL\_CELL\_CARCINOMA |
| Enrichment Score (ES) | -0.2465806 |
| Normalized Enrichment Score (NES) | -0.9264125 |
| Nominal p-value | 0.57641196 |
| FDR q-value | 0.7795261 |
| FWER p-Value | 1.0 |
Table: GSEA Results Summary

  

Fig 1: Enrichment plot: KEGG\_BASAL\_CELL\_CARCINOMA      
 Profile of the Running ES Score & Positions of GeneSet Members on the Rank Ordered List

  

| SYMBOL | TITLE | RANK IN GENE LIST | RANK METRIC SCORE | RUNNING ES | CORE ENRICHMENT || 1 | FZD1 | frizzled class receptor 1 [Source:HGNC Symbol;Acc:HGNC:4038] | 111 | 0.898 | 0.0545 | No |
| 2 | FZD7 | frizzled class receptor 7 [Source:HGNC Symbol;Acc:HGNC:4045] | 274 | 0.768 | 0.0959 | No |
| 3 | WNT5B | Wnt family member 5B [Source:HGNC Symbol;Acc:HGNC:16265] | 327 | 0.734 | 0.1435 | No |
| 4 | FZD2 | frizzled class receptor 2 [Source:HGNC Symbol;Acc:HGNC:4040] | 1111 | 0.494 | 0.1172 | No |
| 5 | WNT5A | Wnt family member 5A [Source:HGNC Symbol;Acc:HGNC:12784] | 1995 | 0.361 | 0.0739 | No |
| 6 | WNT3 | Wnt family member 3 [Source:HGNC Symbol;Acc:HGNC:12782] | 2003 | 0.360 | 0.0987 | No |
| 7 | DVL2 | dishevelled segment polarity protein 2 [Source:HGNC Symbol;Acc:HGNC:3086] | 2693 | 0.280 | 0.0648 | No |
| 8 | WNT2 | Wnt family member 2 [Source:HGNC Symbol;Acc:HGNC:12780] | 2700 | 0.279 | 0.0840 | No |
| 9 | GLI3 | GLI family zinc finger 3 [Source:HGNC Symbol;Acc:HGNC:4319] | 2861 | 0.264 | 0.0901 | No |
| 10 | WNT2B | Wnt family member 2B [Source:HGNC Symbol;Acc:HGNC:12781] | 4194 | 0.138 | -0.0038 | No |
| 11 | DVL1 | dishevelled segment polarity protein 1 [Source:HGNC Symbol;Acc:HGNC:3084] | 4612 | 0.104 | -0.0289 | No |
| 12 | WNT11 | Wnt family member 11 [Source:HGNC Symbol;Acc:HGNC:12776] | 4688 | 0.097 | -0.0279 | No |
| 13 | TCF7L2 | transcription factor 7 like 2 [Source:HGNC Symbol;Acc:HGNC:11641] | 5034 | 0.070 | -0.0499 | No |
| 14 | FZD5 | frizzled class receptor 5 [Source:HGNC Symbol;Acc:HGNC:4043] | 5392 | 0.041 | -0.0748 | No |
| 15 | TP53 | tumor protein p53 [Source:HGNC Symbol;Acc:HGNC:11998] | 5511 | 0.032 | -0.0817 | No |
| 16 | WNT6 | Wnt family member 6 [Source:HGNC Symbol;Acc:HGNC:12785] | 5781 | 0.010 | -0.1020 | No |
| 17 | FZD8 | frizzled class receptor 8 [Source:HGNC Symbol;Acc:HGNC:4046] | 6084 | -0.011 | -0.1247 | No |
| 18 | WNT7B | Wnt family member 7B [Source:HGNC Symbol;Acc:HGNC:12787] | 6230 | -0.023 | -0.1344 | No |
| 19 | FZD9 | frizzled class receptor 9 [Source:HGNC Symbol;Acc:HGNC:4047] | 6812 | -0.064 | -0.1751 | No |
| 20 | WNT7A | Wnt family member 7A [Source:HGNC Symbol;Acc:HGNC:12786] | 7166 | -0.093 | -0.1960 | No |
| 21 | APC2 | APC regulator of WNT signaling pathway 2 [Source:HGNC Symbol;Acc:HGNC:24036] | 7271 | -0.101 | -0.1970 | No |
| 22 | TCF7L1 | transcription factor 7 like 1 [Source:HGNC Symbol;Acc:HGNC:11640] | 7383 | -0.109 | -0.1980 | No |
| 23 | LEF1 | lymphoid enhancer binding factor 1 [Source:HGNC Symbol;Acc:HGNC:6551] | 7467 | -0.116 | -0.1963 | No |
| 24 | WNT10B | Wnt family member 10B [Source:HGNC Symbol;Acc:HGNC:12775] | 7700 | -0.134 | -0.2049 | No |
| 25 | BMP4 | bone morphogenetic protein 4 [Source:HGNC Symbol;Acc:HGNC:1071] | 7816 | -0.142 | -0.2039 | No |
| 26 | FZD4 | frizzled class receptor 4 [Source:HGNC Symbol;Acc:HGNC:4042] | 8159 | -0.168 | -0.2187 | No |
| 27 | FZD10 | frizzled class receptor 10 [Source:HGNC Symbol;Acc:HGNC:4039] | 8354 | -0.182 | -0.2210 | No |
| 28 | FZD6 | frizzled class receptor 6 [Source:HGNC Symbol;Acc:HGNC:4044] | 8379 | -0.184 | -0.2099 | No |
| 29 | DVL3 | dishevelled segment polarity protein 3 [Source:HGNC Symbol;Acc:HGNC:3087] | 8756 | -0.213 | -0.2242 | Yes |
| 30 | WNT16 | Wnt family member 16 [Source:HGNC Symbol;Acc:HGNC:16267] | 8808 | -0.218 | -0.2129 | Yes |
| 31 | PTCH1 | patched 1 [Source:HGNC Symbol;Acc:HGNC:9585] | 8837 | -0.220 | -0.1996 | Yes |
| 32 | SHH | sonic hedgehog signaling molecule [Source:HGNC Symbol;Acc:HGNC:10848] | 8941 | -0.227 | -0.1917 | Yes |
| 33 | PTCH2 | patched 2 [Source:HGNC Symbol;Acc:HGNC:9586] | 8994 | -0.231 | -0.1795 | Yes |
| 34 | FZD3 | frizzled class receptor 3 [Source:HGNC Symbol;Acc:HGNC:4041] | 9560 | -0.279 | -0.2038 | Yes |
| 35 | SMO | "smoothened, frizzled class receptor [Source:HGNC Symbol;Acc:HGNC:11119]" | 10111 | -0.332 | -0.2232 | Yes |
| 36 | AXIN1 | axin 1 [Source:HGNC Symbol;Acc:HGNC:903] | 10335 | -0.354 | -0.2157 | Yes |
| 37 | APC | APC regulator of WNT signaling pathway [Source:HGNC Symbol;Acc:HGNC:583] | 10440 | -0.366 | -0.1981 | Yes |
| 38 | WNT1 | Wnt family member 1 [Source:HGNC Symbol;Acc:HGNC:12774] | 10706 | -0.394 | -0.1910 | Yes |
| 39 | BMP2 | bone morphogenetic protein 2 [Source:HGNC Symbol;Acc:HGNC:1069] | 10919 | -0.420 | -0.1780 | Yes |
| 40 | GSK3B | glycogen synthase kinase 3 beta [Source:HGNC Symbol;Acc:HGNC:4617] | 11157 | -0.451 | -0.1647 | Yes |
| 41 | GLI2 | GLI family zinc finger 2 [Source:HGNC Symbol;Acc:HGNC:4318] | 11195 | -0.456 | -0.1355 | Yes |
| 42 | WNT8B | Wnt family member 8B [Source:HGNC Symbol;Acc:HGNC:12789] | 11532 | -0.505 | -0.1262 | Yes |
| 43 | WNT4 | Wnt family member 4 [Source:HGNC Symbol;Acc:HGNC:12783] | 12331 | -0.686 | -0.1400 | Yes |
| 44 | GLI1 | GLI family zinc finger 1 [Source:HGNC Symbol;Acc:HGNC:4317] | 12333 | -0.687 | -0.0918 | Yes |
| 45 | TCF7 | transcription factor 7 [Source:HGNC Symbol;Acc:HGNC:11639] | 12628 | -0.813 | -0.0576 | Yes |
| 46 | CTNNB1 | catenin beta 1 [Source:HGNC Symbol;Acc:HGNC:2514] | 12866 | -1.119 | 0.0026 | Yes |
Table: GSEA details [plain text format]

  

Fig 2: KEGG\_BASAL\_CELL\_CARCINOMA      
 Blue-Pink O' Gram in the Space of the Analyzed GeneSet

  

Fig 3: KEGG\_BASAL\_CELL\_CARCINOMA: Random ES distribution      
 Gene set null distribution of ES for **KEGG\_BASAL\_CELL\_CARCINOMA**
